# Underestimated incidence of kidney disease in nonrenal outpatient

**DOI:** 10.1080/0886022X.2017.1279551

**Published:** 2017-01-24

**Authors:** Zeyuan Lu, Jianyong Yin, Guangyuan Zhang, Rui Wu, Qing Zhao, Niansong Wang, Chungen Yan, Feng Wang

**Affiliations:** a Department of Nephrology, Shanghai Jiao Tong University Affiliated Sixth People’s Hospital, Shanghai, China;; b Department of Urology, Zhongda Hospital, Southeast University, Nanjing, China;; c Department of Cardiology, Shanghai Jiao Tong University Affiliated Sixth People?s Hospital, Shanghai, China;; d Department of Internal Medicine, Medical School of Shaoxing Univerisity, Shaoxing, China;; e Department of Nephrology, Shanghai Eighth People's Hospital, Shanghai, China

**Keywords:** Kidney injury, chronic kidney disease, hypertension, diabetes mellitus, coronary disease

## Abstract

**Background and aims:** Chronic kidney disease (CKD) has been regarded as a severe threaten to public health, a large percentage of CKD are secondary to other diseases. Serum creatinine is the most common marker of renal function, but it did not always reflect glomerular filtration rate (GFR) accurately. In order to investigate the prevalence of kidney disease in non-renal departments and to provide a basis for the prevention of kidney injury, the present study was conducted in several medical centers.

**Methods:** A total of 17,462 outpatients were selected randomly from the departments of cardiology, endocrinology, and neurology in 16 hospitals and the incidence of kidney disease was screened. Estimated GFR (eGFR) was calculated by using MDRD-formula.

**Results:** There are 5293 (30.1%) patients’ eGFR above 90 mL/min/1.73m^2^ among all the subjects in non-renal departments, and 4055(23%) patients’ eGFR lower than 60 mL/min/1.73 m^2^ including 80 patients whose eGFR were below 15 mL/min/1.73 m^2^. Furthermore, among 16616 subjects who have a normal SCr level, there are 3209 respondents’ eGFR lower than 60 mL/min/1.73 m^2^. Moreover, individuals with hypertension or diabetes had a high prevalence of decreased renal function.

**Conclusions:** This survey indicated kidney injury wildly existed in non-renal outpatients, and the incidence of CKD is underestimated.

## Introduction

Accompanied by the demographic of the outpatient population changing, individuals suffer from preexisting kidney injury grow increasingly.^1^ Kidney injury, including acute kidney injury (AKI) and chronic kidney disease (CKD), has become an urgent important problem for public health. Many epidemiological studies have revealed significant associations of kidney injury with high mortality and medical costs. Recently, CKD incidence globally grew at an unexpected rate, partly because the main causes of CKD, including diabetes mellitus and hypertension, have an increased incidence due to obesity, poor nutrition, and lack of exercise.^2^

Kidney disease is known to be associated with other diseases. Risk factors for CKD include age (more than 60 years), hypertension, diabetes, cardiovascular disease, and a family history of the renal disease. Meanwhile, several studies have reported that kidney dysfunction, even at early stages, is an important independent risk factor for many other diseases and death.^3^ Additionally, kidney disease is increasingly recognized as a cause of morbidity among persons living with hypertension, diabetes mellitus, and other cardiovascular diseases.

Since kidney injury is closely associated with many other diseases, the morbidity of kidney injury in non-nephrology departments is underestimated. Although most patients with early stages of chronic kidney disease are asymptomatic, kidney injury is being recognized by nephrologists as an important health problem for a long time.^4^ However, renal function, in many cases, is not carefully examined by specialists in other fields. Considering special prescriptions should be applied in these renal injury patients, we have to figure out the real incidence of kidney disease in non-nephrology outpatient. In order to investigate the prevalence and related risk factors for kidney disease in different hospital departments, and to provide a basis for the prevention of kidney injury, we performed a large scale of survey in hospitals.

## Methods

The study was approved by Ethical Committee of Shanghai Jiao Tong University Affiliated Sixth People's Hospital and written informed consent was obtained from all subjects. From 1 December 2015 to 29 February 2016, 17462 subjects selected from the departments of cardiology, endocrinology, and neurology in sixteen hospitals were screened for kidney injury. Actually, our study was conducted widely in given departments of different medical center. Patients are selected randomly from different medical center. After the obtained patients’ consent, the survey includes medical records and other basic data were collected. When they are qualified our inclusion criteria, their fasting venous blood was collected in the morning for measurement. Inclusion criteria was listed as below: (1) outpatients with diagnosed related disease; (2) no kidney disease history. These individuals visited hospitals for hypertension, diabetes mellitus, and other non-renal diseases.

Creatinine Assay Kit (Kehua, Shanghai, China) was used for serum creatinine measurement. Clinical data, serum creatinine, and the estimated glomerular filtration rate (eGFR) were calculated. Kidney function was assessed by using eGFR, which was determined according to the simplified Modification of Diet in Renal Disease (MDRD) formula: eGFR is equal to 186 × Scr^−1.154 ^×^ ^age^−0.203^× (0.742 if female).^5^ Chronic kidney disease was diagnosed according to the CKD criteria as the presence of kidney damage or decreased kidney function for ≥3 months.^6^ K/DOQI CKD Guidelines (The National Kidney Foundation’s classification) was used for staging of eGFR and classification of CKD.^7^ Hypertension was defined as systolic pressure ≥140 mmHg and diastolic pressure ≥90 mmHg. Diabetes mellitus type 2 is a type of diabetes that is characterized by hyperglycemia, insulin resistance, and obesity. Coronary artery disease (CAD) was diagnosed when stenosis was evident in ≥50% of the luminal diameter of a major coronary artery, that is, the left main coronary artery, left anterior descending artery or its first diagonal branch, left circumflex artery or its first obtuse marginal branch, or right coronary artery. None of the patients involved in the study has been previously tested as early renal damage.

Normally distributed data are expressed as the mean standard deviation; groups were compared using two independent sample *t*-tests or analysis of variance (ANOVA). Nonparametric data are expressed as medians (25–75% interquartile range). A two-sided *p* values <0.05 was considered significant.

## Results

There are 18,007 patients enrolled in the research from 2015 to 2016. A total of 17,462 individuals were included in the final analysis. Patients were excluded for missing data or known kidney disease. Overall, the study included 17,462 patients, mean age of 67.6 ± 13 years, of whom 8285 (47.4%) were females, mean age 68.1 ± 12.9, and 9177 (52.6%) male, mean average age 67.1 ± 13.1. 11,035 (59.5%) respondents were older than 65 years. Besides, more than half of respondents (54.6%) developed hypertension. Hypertension with type-2 DM was confirmed in 18.3% of patients, while those who suffered from type-2 DM isolated were present in 22.6% cases. Additionally, there are 257 (1.5%) patients suffer from coronary heart disease according to the datum.

**Figure 1.**
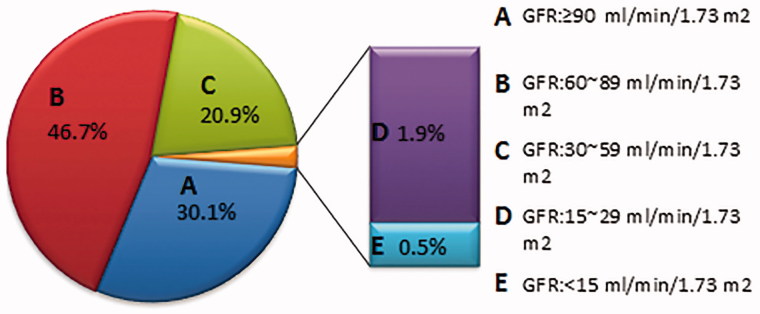
The distribution of the patients’ estimated GFR.

In all respondents, there are 4055 (23%) patients’ eGFR lower than 60 mL/min/1.73 m^2^ including 80 patients whose eGFR below 15 mL/min/1.73 m^2^. Besides, patients who have eGFR between 6 0 ∼ 90 mL/min/1.73 m^2^ have the largest population. There are 8185 (46.7%) patients in that range, almost account for half of the respondents. Furthermore, there are only 5256 (30.1%) patients’ eGFR above 90 mL/min/1.73 m^2^ (Figure 1).

**Figure 2.**
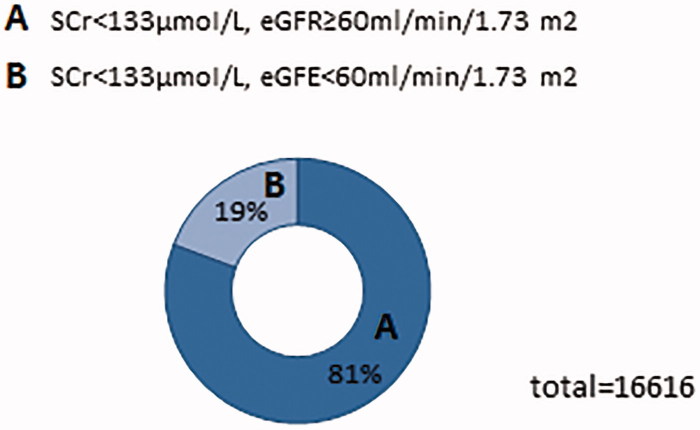
Proportions of patients’ abnormal eGFR in general.

Among 16,616 subjects who have a normal SCr level, there are 3209 respondents’ eGFR lower than 60 mL/min/1.73 m^2^. Different kinds of diseases have different proportions (Table 1) (Figure 2). Normal creatinine levels range from 0.7 to 1.3 mg/dL in men and 0.6 to 1.1 mg/dL in women. In general, the normal range of creatinine level is 44–106 μmol/L.

**Table 1.** Proportions of patients’ number with normal SCr and abnormal eGFR in high-risk diseases.

Group	eGFR <60 (mL/min/1.73 m^2^)	eGFR <60, SCr <133 (mL/min/1.73 m^2^)
Patients over 65 years, *n* = 11035	3377 (31%)	2697 (24%)
Patients with hypertension, *n* = 9542	2092 (22%)	1696 (18%)
Patients with diabetes, *n* = 3938	787 (20%)	636 (16%)
Patients with coronary disease, *n* = 257	62 (24%)	49 (19%)
Patients with stroke, *n* = 179	27 (15%)	21 (12%)
Patients with hypertension and diabetes, *n* = 3189	990 (31%)	731 (23%)
Patients with hypertension and coronary disease, *n* = 237	62 (26%)	51 (22%)
Patients with diabetes and coronary diabetes, *n* = 22	7 (32%)	7 (32%)
Patients with hypertension, diabetes and coronary disease, *n* = 39	23 (59%)	18 (46%)
Patients with other disease, *n* = 59	10 (17%)	5 (8%)

**Figure 3.**
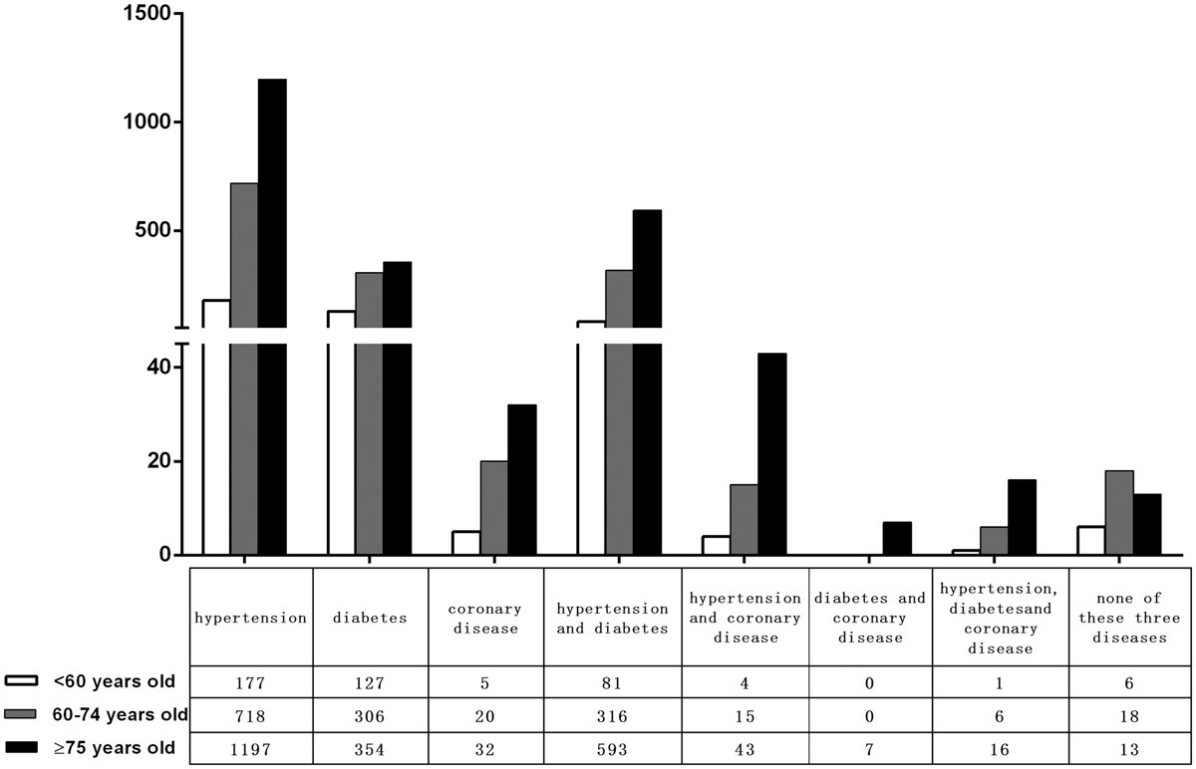
Complications of the patients with kidney injury.

Individuals with hypertension or diabetes had a high prevalence of decreased kidney function (Figure 3). As for hypertensive patients, with the greatest prevalence of decreased kidney function, possibly reflecting increased severity or duration of hypertension in these individuals. Moreover, the figure for low eGFR respondents gradually increased with age.

## Discussion

Kidney injury, includingAKI and CKD, is a severe and common clinical syndrome with adverse outcome and it becomes a worldwide public health problem.^8–10^ As reported there are over 1.1 million patients maintain dialysis in worldwide, and the size of this population has been expanding at a rate of 7% per year.^11^ In additional to the high incidence of CKD in public, it has been demonstrated that prevalence was higher in hypertension, diabetes, and older age.^12^ Also, cardiovascular disease (CVD) is associated with subsequent kidney function decline and development of kidney disease. Another reason is widely utilized contrast agents in clinical diagnostic and interventional procedures, especially in patients with heart disease, while these contrast agents can also lead contrast-induced nephropathy (CIN), which is one of the leading causes of hospital-acquired acute kidney injury.^13^ Therefore, evaluation and management of these diseases is important for patients’ well-being and may improve the course of CKD. This is particularly important for patients with diabetes and high blood pressure, the leading causes of chronic kidney disease and cardiovascular disease in many countries.

In this study, we investigated the renal function in 17,462 non-nephrology outpatients. Firstly, compared with the reported incidence of kidney injury in public, the proportion of the renal injury is relatively higher in the fields of cardiology, endocrinology, and neurology. This phenomenon is particularly remarkable among respondents with both hypertension and diabetes. Previous studies had indicated that hypertension, diabetes, and CKD interacted to each other. They also confirm that early chronic kidney disease is high prevalence in high-risk outpatients in Sarajevo.^4^ This finding is consistent with our results. However, except those high-risk outpatients, we also investigate outpatients in different fields. It means this study have varieties basic diseases. Moreover, the factor of age is also taken into consideration. Secondly, in addition to conforming non–nephrology outpatients have a high prevalence of kidney injury, we also indicated that doctors have low awareness of patients’ kidney injury. Many other studies have similar conclusions. One of the articles indicated that CKD awareness in the U.S. population is low, even though it has a high prevalence.^12^ Another study proved that individuals with coronary heart disease (CHD) have a high prevalence of CKD in US population, and most of these individuals were unaware of their of their kidney disease.^14^ Thirdly, according to our outcome, the SCr level cannot fully represent the kidney function actually. Many people with normal SCr also have an abnormal eGFR level. Those are important phenomenon since these underlying renal diseases should be considered for the treatment.

Our study has some limitations as well. One is that some data were missing since it is difficult to trace outpatient clinic information. However, the sample size is large enough, thus the result is reliable. Another limitation is that our research only covered several departments, and hence, outpatients that we random chosen cannot represent all kinds of basic diseases patients, and as a result, it may lead to outcome bias. In addition, only once measure was not enough for steady-state assessment for patient. There may be sampling error existed. However, as large sample size enough, we still believe that the sample error was minimized and our results are reliable.

Among patients with cardiovascular or endocrine diseases, the high prevalence of kidney injury and general underestimated by clinicians has important implications. For the sake of safety treatment, physicians should avoid overlooking kidney injury in those non-nephrology outpatients. Since the serum creatinine cannot reflect kidney function accurately, physicians who rely on serum creatinine to identify underlying kidney disease will under-diagnose the presence of impaired kidney function. Physicians suggested be educated that estimated GFR should be measured, no matter whether SCr level is normal.^15^ It means that physicians in non-nephrology departments should also pay close attention to kidney function even the SCr is normal. Recommendations for evaluating people at increased risk are to measure urine albumin to assess kidney damage and to estimate the GFR with an equation based on the level of SCr.^16^^,^^17^ Taking into consideration, majority of patients should routinely have an eGFR measuring for the presence of kidney disease.

As for outpatients, leading to lifestyle modifications and risk factors treatments are necessary for preventing progressive kidney diseases.^14^ We should show solicitude for the clinical decisions to manage the kidney related diseases. Cardiovascular disease is singled out from among the possible comorbid conditions to emphasize its complex relationship with chronic kidney disease, and its importance as a preventable cause of morbidity and mortality in patients with chronic kidney disease. Blood pressure management of patients with kidney disease should lower target goal of 130/80 mmHg and recommend to use either an angiotensin-converting enzyme inhibitor or receptor blocker. Dietary protein should be limited to 0.8 g/kg/day.^18^ What is more, kidney disease should alert patients to reduce exposure to nephrotoxins like radiographic dye and nonsteroidal anti-inflammatory agents and to review medications for possible renal dosing modifications.^14^ There are also published guideline give suggestions about how to deal with kidney injury accompanied many other diseases.^19^

## Conclusions

Chronic kidney disease has a high prevalence in the non-nephrology departments. However, this incidence is underestimated by not only the general public but also clinicians. Since renal injury can directly affect therapeutic schedules, non-nephrology physicians should pay close attention to kidney function.
